# Growth Mechanism of Semipolar AlN Layers by HVPE on Hybrid SiC/Si(110) Substrates

**DOI:** 10.3390/ma15186202

**Published:** 2022-09-06

**Authors:** Alexander A. Koryakin, Sergey A. Kukushkin, Andrey V. Osipov, Shukrillo Sh. Sharofidinov, Mikhail P. Shcheglov

**Affiliations:** 1 Faculty of Physics, St. Petersburg State University, St. Petersburg 199034, Russia; 2 Institute for Problems in Mechanical Engineering of the Russian Academy of Sciences, St. Petersburg 199178, Russia; 3 Ioffe Institute Russian Academy of Sciences, St. Petersburg 194021, Russia

**Keywords:** semipolar aluminum nitride, A3B5 compounds, silicon carbide on silicon, HVPE

## Abstract

In this work, the growth mechanism of aluminum nitride (AlN) epitaxial films by hydride vapor phase epitaxy (HVPE) on silicon carbide (SiC) epitaxial layers grown on silicon (110) substrates is investigated. The peculiarity of this study is that the SiC layers used for the growth of AlN films are synthesized by the method of coordinated substitution of atoms. In this growth method, a part of the silicon atoms in the silicon substrate is replaced with carbon atoms. As a result of atom substitution, the initially smooth Si(110) surface transforms into a SiC surface covered with octahedron-shaped structures having the SiC(111) and SiC($11\bar{1}$) facets. The SiC(111)/($11\bar{1}$) facets forming the angle of 35.3° with the original Si(110) surface act as “substrates” for further growth of semipolar AlN. The structure and morphology of AlN films are investigated by X-ray diffraction (XRD), scanning electron microscopy (SEM), reflection high-energy electron diffraction (RHEED) and Raman spectroscopy. It is found that the AlN layers are formed by merged hexagonal microcrystals growing in two directions, and the following relation is approximately satisfied for both crystal orientations: AlN($10\bar{1}3$)||Si(110). The full-width at half-maximum (FWHM) of the X-ray rocking curve for the AlN($10\bar{1}3$) diffraction peak averaged over the sample area is about 20 arcmin. A theoretical model explaining the presence of two orientations of AlN films on hybrid SiC/Si(110) substrates is proposed, and a method for controlling their orientation is presented.

## 1. Introduction

The study of the growth mechanisms of epitaxial AlN films is important for several reasons. Firstly, wurtzite AlN is a direct-gap semiconductor with a large band gap (6.2 eV) and therefore is a promising material for the manufacturing of ultraviolet optoelectronic devices [1]. Secondly, thin AlN layers are widely used as buffer layers for GaN growth [2]. For example, the formation of thin AlN layers on 6H–SiC substrates before GaN growth allows the realization of a layer-by-layer growth mode with higher probability than a three-dimensional growth mode. Moreover, during GaN growth on silicon substrates, gallium can chemically interact with silicon, leading to a significant degradation of the film; however, the deposition of AlN layers before the growth prevents this process. Most studies of epitaxial growth of III-nitride films are focused on the growth of polar layers [3]. However, in this case, large electric fields arise in the III-nitride film due to the piezoelectric effect and spontaneous polarization that cause the deterioration of device characteristics. It was found [4] that the intensity of polarization effects strongly depends on the crystallographic orientation of the film and can be significantly reduced by synthesizing nonpolar and semipolar III-nitride layers. Therefore, the development of optoelectronic devices based on nonpolar and semipolar layers of III-nitrides is of high importance. At present, there are very few investigations of the initial growth stage of semipolar AlN layers, because the technology of semipolar AlN layer synthesis is complex. A detailed description of the complicated preparation techniques of substrates for the growth of semipolar AlN and GaN films can be found in the review [5]. Nevertheless, semipolar AlN layers have been obtained using metalorganic vapor-phase epitaxy (MOVPE) [6,7,8], hydride vapor phase epitaxy (HVPE) [5,9,10,11,12], plasma assisted molecular beam epitaxy (PA MBE) [13] and pulsed laser deposition [14]. Growth of semipolar AlN has been demonstrated on Al_2_O_3_($10\bar{1}0$) substrates (m-plane) [7,8,10,13], SiC/Si(100) templates [6,9,11,12] and ZnO($1\bar{1}02$) (r-plane) [14]. In the previous works [5,9,11,12], hybrid substrates consisting of 3C–SiC epitaxial layers synthesized on a vicinal silicon surface deviated from the Si(100) plane by 4°÷7° were used for the growth of AlN and GaN layers. The synthesis of the 3C–SiC epitaxial layer was performed by the method of coordinated substitution of atoms. This method involves a chemical treatment of the surface of silicon substrate in the CO gas atmosphere. A detailed description of the method and the growth conditions to synthesize the hybrid SiC/Si substrates can be found in the reviews [15,16].

The advantages of the use of the hybrid SiC/Si substrates grown by the method of coordinated substitution of atoms for the growth of semipolar III-nitrides as compared to templates obtained by other methods are as follows. Usually, in order to grow semipolar III-nitride layers on various substrates, special etching techniques must be used to form the wedge-shaped facets of specific orientation on which semipolar III-nitrides could nucleate [5]. However, in the method of coordinated substitution of atoms, the wedge-shaped structure formation occurs naturally. It was shown in [5] that, during SiC synthesis on Si(100), the vicinal steps on the Si(100) plane are transformed into an array of parallel triangular-shaped SiC prisms faceted by the SiC(111) planes. Since the symmetry of atomic arrangement on the surfaces of such prisms is characteristic of both cubic and hexagonal crystals, crystals with both cubic and hexagonal symmetry can grow on their surfaces. The stable crystal phase will not be determined by the orientation of the substrate, but only by the growth conditions, i.e., temperature and incident fluxes. This opens up new perspectives for the growth of hexagonal semipolar crystals.

According to the theoretical results obtained in [15], the Si(110) surface during the synthesis of the hybrid SiC/Si(110) substrate will transform into the (110)-oriented surface covered with octahedron-like structures, one side of which is the SiC(111) facet and the other is the SiC($11\bar{1}$) facet. These growth figures merge with each other forming “mountain ranges” separated by “depressions”. The plane between the “mountain ranges” is the SiC(110) plane. The remaining planes of the SiC{111} and SiC{$11\bar{1}$} families are perpendicular to the Si(110) plane. The nucleation of semipolar AlN layers will occur primarily on the surface of these SiC facets. As a result, the AlN crystals oriented in two directions will grow on the surface of the SiC/Si(110) substrate, and the following crystallographic relation should be satisfied: AlN(0001)/($000\bar{1}$)||SiC(111)/($11\bar{1}$). The nucleation of AlN on the SiC(110) facets will occur with a lower probability and only at high values of supersaturation; therefore, this process will occur much more slowly. The aim of this report is to prove experimentally the assumptions concerning the growth of AlN on the hybrid SiC/Si(110) substrates and to perform a rigorous quantitative analysis of the initial growth stage of AlN by HVPE on these substrates. The paper is organized as follows. In Section 2, the experimental details of AlN film synthesis on the hybrid SiC/Si(110) substrates are described together with the growth conditions for the synthesis of SiC layers on Si(110) substrates. In Section 3, the results of investigations of the morphology and structure of AlN/SiC/Si(110) system are presented. Particularly, the presence of two crystallographic orientations of AlN films are experimentally proven, and it is shown that the following crystallographic relation is approximately satisfied: AlN($10\bar{1}3$)||Si(110). In Section 4, a model of AlN nucleation on SiC/Si(110) is proposed, which is fully consistent with the experimental data. A method for controlling the orientation of AlN microcrystals is also presented in this section. Summary of our results and conclusions are given in Section 5.

## 2. Materials and Methods

The SiC buffer layer was synthesized by the method of coordinated substitution of atoms [16] in the atmosphere of CO and SiH_4_ on p-Si(110) substrates with a resistivity of 50 Ω∙cm. The SiC growth was performed at 1250 °C for 15 min and involves the following chemical reaction between silicon and carbon monoxide:

(1) $$2\mathrm{Si}\left(\mathrm{crystal}\right)+\mathrm{CO}\left(\mathrm{gas}\right)=\mathrm{SiC}\left(\mathrm{crystal}\right)+\mathrm{SiO}\left(\mathrm{gas}\right)$$

The gas consumption was 12 sccm and 0.25 sccm for CO and SiH_4_, respectively, at the CO pressure of 2 Torr. The silicon substrates were cleaned of the oxide layer before the synthesis and then passivated with hydrogen atoms according to the procedure developed in [17]. After the formation of the SiC layer on the Si(110) substrates, an AlN layer was grown by HVPE. The AlN growth is the result of the following chemical reaction in the growth zone [5]:

(2) $${\mathrm{AlCl}}_{3}\left(\mathrm{gas}\right)+{\mathrm{NH}}_{3}\left(\mathrm{gas}\right)=\mathrm{AlN}\left(\mathrm{crystal}\right)+3\mathrm{HCl}\left(\mathrm{gas}\right)$$

The AlN growth was performed at a temperature of 1080 °C for 5 min. The NH_3_ flux and HCl flux (over a boat with liquid aluminum) were 1 L/min and 0.3 L/min at the Ar flow of 4 L/min, which was a carrier gas. Before the growth of the AlN layer, the SiC/Si(110) substrate was kept in the HCl stream for 3 min at the flow of 0.1 L/min to remove contaminants.

## 3. Results

**Figure 1:**
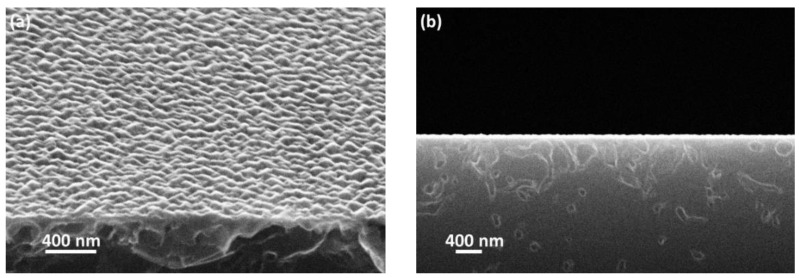
SEM images of a SiC/Si(110) substrate (tilted view (**a**) and side view (**b**)).

**Figure 2:**
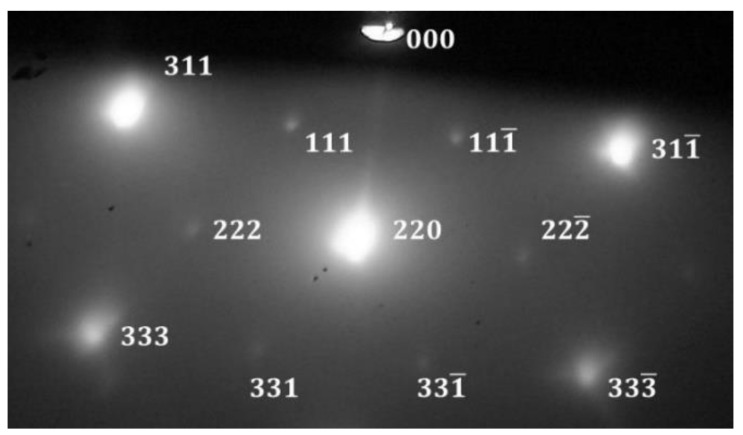
RHEED pattern of a SiC/Si(110) substrate.

Figure 1 shows typical scanning electron microscopy (SEM) images of the SiC/Si(110) substrate. It is seen that the SiC surface with the thickness of ∼70 nm consists of “hills” with the average lateral size of 100 nm and the average height of 20 nm. The pores are formed under the SiC layer as a result of the topochemical reaction (Equation (1)). The inner surface of the pores is covered by a SiC layer. The surface of the SiC layer was also investigated by reflection high-energy electron diffraction (RHEED). Figure 2 shows the RHEED image obtained using EMR-100 electron diffractometer with an electron energy of 50 keV. At such energies, the electron penetration depth in the sample is less than ∼100 nm, and therefore the RHEED pattern corresponds only to the SiC layer. It is seen from Figure 2 that the SiC layer is monocrystalline and consists of the cubic polytype (3C–SiC). The presence of epitaxial spot-like reflexes, SiC(111) and SiC(220), indicates that the surface layer is rough (with “hills”), otherwise the diffraction pattern would consist of streak-like reflexes [18], and also that the facets of these “hills” are the SiC{111}, SiC{$11\bar{1}$} and SiC{110} facets.

**Figure 3:**
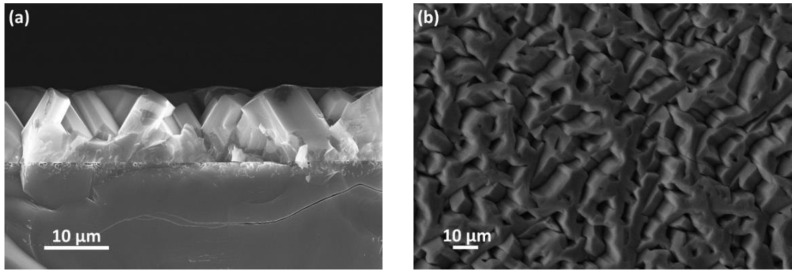
SEM images of the AlN/SiC/Si(110) structure (side view (**a**) and top view (**b**)).

The AlN layers were investigated by SEM and X-ray diffraction (XRD). Figure 3 shows SEM images of an AlN film grown on the SiC/Si(110) substrate. These investigations revealed the following features of the structure and morphology of the AlN layer. It was found that the AlN layer consists of merged hexagonal crystals with a height of ~12 µm. The results of XRD measurements in the θ-2θ mode showed the presence of two symmetrically arranged peaks corresponding to the AlN($10\bar{1}3$) planes. The alignment of AlN hexagonal crystals on the SiC/Si(110) substrate occurs by rotating the *c*-axis by ~30° relative to the substrate normal. The number of hexagonal crystals rotated to one side is two times larger than the number of hexagonal crystals rotated to the other side (according to the intensities of X-ray peaks). The AlN($10\bar{1}3$) reflecting planes of the differently oriented crystals are disoriented relative to each other by 7° in the direction of AlN crystal rotation and by not more than 0.5° in the perpendicular direction. The full-width at half-maximum (FWHM) of the X-ray rocking curves for the differently oriented crystals (the diffraction peak AlN($10\bar{1}3$) averaged over the sample area) is ~20′ for the first case and ~30′ for the second case.

**Figure 4:**
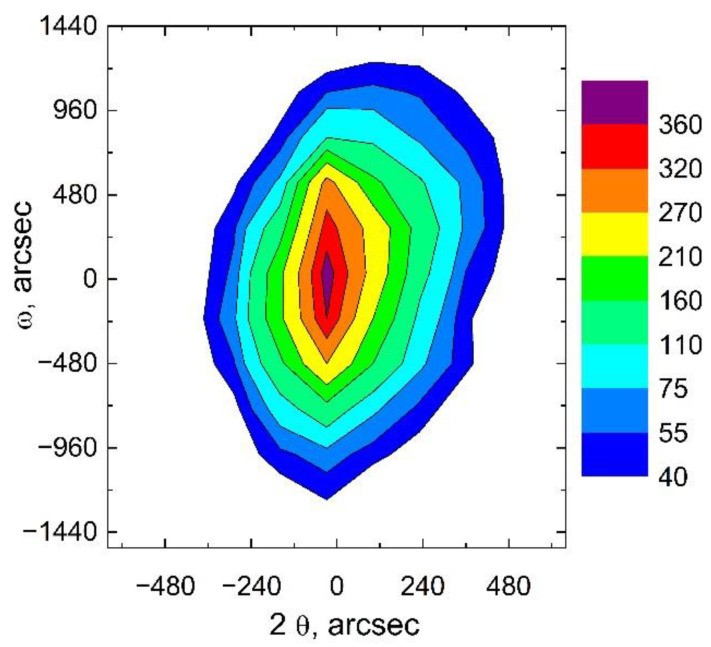
XRD intensity distribution map (in arbitrary units) for the AlN($10\bar{1}3$) symmetric peak. FWHM for this peak is 21′.

The AlN($10\bar{1}3$) XRD peak from the AlN/SiC/Si(110) structure was also analyzed by three-crystal X-ray diffractometry (Figure 4). From the asymmetry of the intensity distribution, one can conclude that there are residual stresses in the AlN layer. The magnitude of residual strain in the AlN layer was estimated by measuring the positions of the XRD peaks for one of the families of differently oriented crystals. It was found that the position of the AlN(0002) and AlN($10\bar{1}3$) peak is θ = 18°00′52″ and θ = 33°01′45″, respectively. Then, the following values of the AlN lattice parameters were calculated: *c* = 0.49815 nm and *a* = 0.31078 nm. This means that the AlN lattice is compressed along the direction of *c*-axis and along the perpendicular direction at a strain of −1 × 10^−4^ and −1.3 × 10^−3^, respectively. The lattice parameters of the relaxed AlN crystal were determined using the reference data [19].

**Figure 5:**
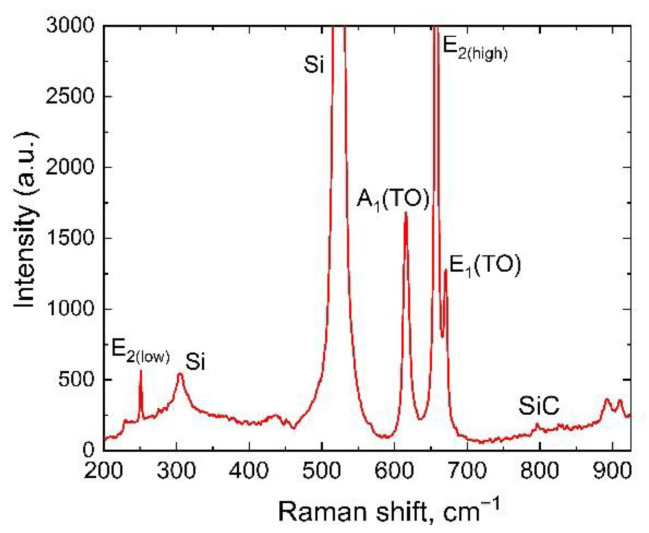
Typical Raman spectrum of the AlN/SiC/Si(110) structure.

Figure 5 shows the Raman spectrum obtained for the AlN/SiC/Si(110) structure using a Witec Alpha 300R micro-Raman microscope with a lateral scanning resolution of ~0.5 μm.

It was found that the AlN peak E_1_(TO) was clearly separated from the AlN main peak E_2_(high), revealing a high crystalline quality of the individual crystallites in the AlN film. It is seen that the E_2_(high) phonon mode has the Raman shift of 657.5 cm^−1^. According to the reference data [20,21], the E_2_(high) phonon mode for unstressed AlN crystals at room temperature have the Raman shift of 657 **÷** 657.4 cm^−1^. Thus, the AlN layer grown on the SiC/Si(110) substrate is almost unstressed, which is consistent with the results of XRD measurements.

## 4. Discussion

Below we develop a theoretical model to explain the presence of two growth directions of AlN crystals on the hybrid SiC/Si(110) substrates and propose an approach for estimating the nucleation rate of AlN crystals growing in these two directions. The experimental data indicate that the SiC/Si(110) surface consists of octahedron-like “hills” faceted by the SiC{111} and SiC{$11\bar{1}$} planes, and that the edges of these “hills” are truncated by the SiC{110} planes. The smallest lattice mismatch between wurtzite AlN and 3C–SiC, of about 1%, is achieved when the AlN(0001)/($000\bar{1}$) plane and the 3C–SiC(111)/($11\bar{1}$) plane are conjugated. Therefore, it is reasonable to assume that the nucleation and growth of AlN islands (AlN nuclei) occur mainly on the SiC(111) and SiC($11\bar{1}$) facets. In this case, the lattice mismatch between the crystals will be minimal. Note that if an AlN nucleus is formed in the vicinity of the intersection line of the SiC(111)/($11\bar{1}$) facet with another low-index facet, for example SiC(110), the first monolayer of the island can also be formed coherently with this facet. Thus, the following preferable sites for nucleation of AlN islands can be distinguished on the SiC/Si(110) substrate: the SiC(111) facet, the SiC($11\bar{1}$) facet and the corner formed by the SiC(111)/($11\bar{1}$) facet and another low-index facet.

**Figure 6:**
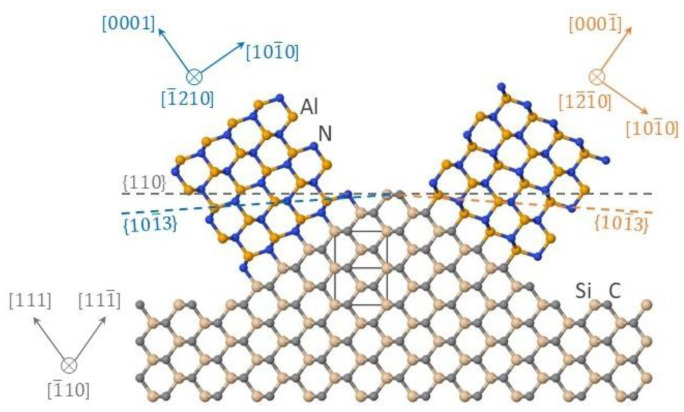
Atomic model of wurtzite AlN nuclei growing in the [0001] and [$000\bar{1}$] directions on a SiC(110) layer. The Al (N) atoms and Si (C) atoms are shown as orange (blue) and beige (grey) spheres, respectively. The SiC unit cell is also shown.

Consider the nucleation of a prism-shaped island with the AlN{$1\bar{1}00$} side walls on the SiC (111) and SiC($11\bar{1}$) facets. It is well known that this island shape is the equilibrium shape of III-nitride crystals [22]. A contact with the substrate only results in a change in the prism height [23]. An atomic model for two possible cases of AlN and SiC lattice conjugation is presented in Figure 6. The reference data [19] were used to define the lattice constants of SiC and AlN. The AlN/SiC interface shown in the figure is ideal; however, the presence of impurity substitution atoms in the AlN lattice or in the SiC lattice at the interface can reduce the interface energy, as was shown in [24,25] (for example, the substitution of a part of Si atoms by Al atoms or a part of C atoms by N atoms or the presence of vacancies). Figure 6 also shows the ideal AlN(0001) and AlN($000\bar{1}$) surfaces for two AlN islands terminated by Al and N atoms, respectively.

The crystallographic orientation of the AlN islands relative to the substrate is given by the following relations: AlN[0001]||SiC[111] and AlN[$\bar{1}210$]||SiC[$\bar{1}10$] (for the nucleus on the left in Figure 6); AlN[000$\bar{1}$]||SiC[$11\bar{1}$] and AlN [$1\bar{2}\bar{1}0$]||SiC[$\bar{1}10$] (for the nucleus on the right). The theoretically predicted value of the angle between the AlN(0001)/($000\bar{1}$) plane and the SiC(110) plane is 35.3° (equal to the angle between the SiC(111)/($11\bar{1}$) plane and the SiC(110) plane). The angle between the AlN($10\bar{1}3$) plane of differently oriented hexagonal crystals and their AlN(0001)/($000\bar{1}$) plane that is parallel to the SiC(111)/($11\bar{1}$) plane is 31.6°. As a result, we find that the AlN($10\bar{1}3$) planes are deviated from the SiC(110) plane by 3.7°. This is in agreement with the experimental data on the angle between the AlN($10\bar{1}3$) planes of the crystals growing in two directions (7°).

According to the classical nucleation theory [23], the nucleation rate of AlN islands is given by the Boltzmann distribution $I\sim \exp\left(-\Delta G^{*}/k_{B}T\right)$, where $\Delta G^{*}$ is the nucleation barrier, $k_{B}$ is the Boltzmann constant and *T* is the growth temperature. Thus, to predict the nucleation probability of AlN islands on the SiC(111) and SiC($11\bar{1}$) facets, firstly, it is necessary to find the Gibbs energy of nucleus formation. The Gibbs energy of three-dimensional AlN island formation is defined by the expression:

(3) $$\Delta G\left(m,n\right)=-3m^{2}n\Delta \mu +3^{3/2}/2m^{2}a^{2}\Delta \gamma +3mnac\gamma_{s}$$

Here, *m* and *n* are the island base radius and the island height expressed in terms of the number of atoms; Δμ is the chemical potential difference per AlN pair; Δγ= $\gamma_{\mathrm{AlN}}+\gamma_{i}-\gamma_{\mathrm{SiC}}$, where $\gamma_{\mathrm{AlN}}$, $\gamma_{\mathrm{SiC}}$ and $\gamma_{i}$ are the surface energy of the AlN(0001)/($000\bar{1}$) facet of the island, the surface energy of the SiC(111)/($11\bar{1}$) facet and the interphase energy of the AlN/SiC(111) interface (or the AlN/SiC($11\bar{1}$) interface); $\gamma_{s}$ is the surface energy of the AlN($1\bar{1}$00) facet. The nucleation barrier of a three-dimensional AlN island equals

(4) $$\Delta G^{*}=\frac{3^{3/2}a^{4}c^{2}\gamma_{s}^{2}\Delta \gamma }{2\Delta \mu^{2}}$$

It follows from Formula (4) that the film growth regime depends on the value of the surface energy difference Δγ, which includes only the energies $\gamma_{\mathrm{AlN}}$, $\gamma_{i}$ and $\gamma_{\mathrm{SiC}}$ (the surface energy of the island side facets $\gamma_{s}$ is not included) [23]. If Δγ  > 0, a three-dimensional growth mode is energetically favorable. If Δγ < 0, a two-dimensional layer is formed, since the film will wet the substrate.

Let us also give the expressions for the Gibbs energy and nucleation barrier of a two-dimensional AlN island:

(5) $$\Delta G\left(m\right)=-3m^{2}\Delta \mu +3^{3/2}/2m^{2}a^{2}\Delta \gamma +3mac\gamma_{s}$$

(6) $$\Delta G^{*}=\frac{3/4\;a^{2}c^{2}\gamma_{s}^{2}}{\Delta \mu -3^{1/2}a^{2}\Delta \gamma /2}$$

where the approximate value of the interfacial energy of the island perimeter (per unit length), $\gamma_{s}$*c*/2, is used. By equating Formulas (4) and (6), it is easy to show that the nucleation barriers for three- and two-dimensional islands are equal when Δμ = 3^1/2^*a*^2^ Δγ provided that Δγ > 0 [23].

The ratio of the nucleation probabilities of the three-dimensional nuclei on SiC(111) and SiC($11\bar{1}$) is given by the following expression:

(7) $$\frac{P_{\left(111\right)}}{P_{\left(11\bar{1}\right)}}\approx \frac{\exp\left(-\Delta G_{\left(111\right)}^{*}/k_{B}T\;\right)}{\exp\left(-\Delta G_{\left(11\bar{1}\right)}^{*}/k_{B}T\;\right)}=\exp\left(-\frac{3^{3/2}a^{4}c^{2}\gamma_{s}^{2}\left(\Delta \gamma_{\left(111\right)}-\Delta \gamma_{\left(11\bar{1}\right)}\right)}{2\Delta \mu^{2}k_{B}T}\right)$$

$\Delta G_{\left(111\right)}^{*}$ and $\Delta G_{\left(11\bar{1}\right)}^{*}$ are the nucleation barriers on the SiC(111) and SiC($11\bar{1}$) facets, $\Delta \gamma_{\left(111\right)}$ and $\Delta \gamma_{\left(11\bar{1}\right)}$ are the surface energy differences for nucleation on SiC(111) and SiC($11\bar{1}$). The expression (7) is obtained by neglecting the difference in the attachment rates of III–V pairs to the AlN islands on SiC(111) and SiC($11\bar{1}$). It follows from Formula (7) that the nucleation rates on these two facets are equal when $\Delta \gamma_{\left(111\right)\;}$ = $\;\Delta \gamma_{\left(11\bar{1}\right)}$. If $\Delta \gamma_{\left(111\right)}$ > $\;\Delta \gamma_{\left(11\bar{1}\right)}$, then the nucleation rate is lower on the SiC(111) facet and vice versa; if $\Delta \gamma_{\left(111\right)}$ < $\;\Delta \gamma_{\left(11\bar{1}\right)}$, then the nucleation rate is higher on the SiC(111) facet. Considering the crystallographic orientations of AlN crystals shown in Figure 6 and using the reference data [24,26,27], we find that the difference $\Delta \gamma_{\left(111\right)}-\Delta \gamma_{\left(11\bar{1}\right)}$ is mainly contributed by the surface energy differences of AlN and SiC, $\Delta \gamma_{\mathrm{AlN}}$ and $\Delta \gamma_{\mathrm{SiC}}$, whereas the energy difference of the AlN/SiC interfaces, $\Delta \gamma_{i}$, is relatively small. A simple thermodynamic analysis is presented below to show how the growth direction of AlN crystals can be controlled.

**Figure 7:**
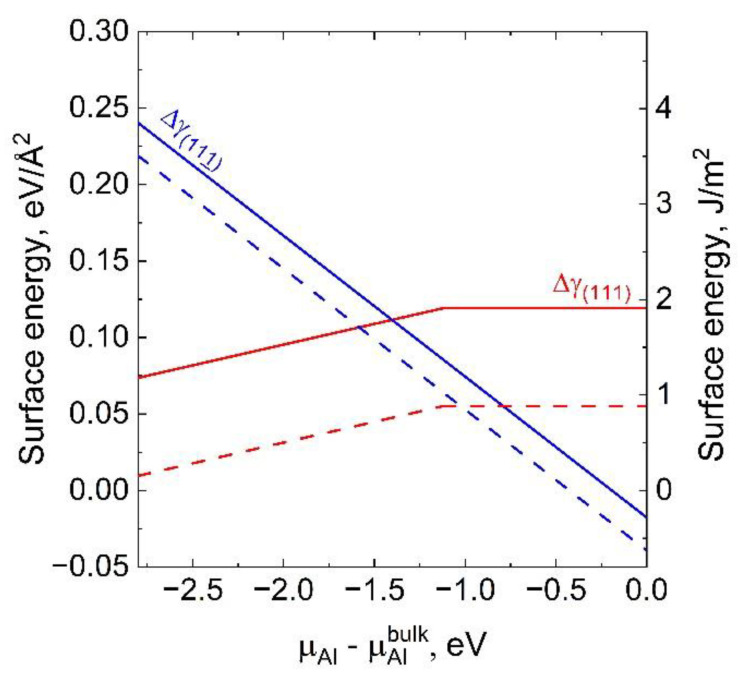
The surface energy difference Δγ as a function of the chemical potential of Al. The results of calculating Δγ using the surface energy of SiC(111) and SiC($11\bar{1}$) [27] for relaxed surfaces (solid lines) and unrelaxed surfaces (dashed lines) are shown. The $\Delta \gamma_{\left(111\right)}$ dependences are calculated using the AlN/SiC(111) interface energy dependence for the case of ideal interface (the increasing parts of the curves) and for the ideal interface in which one Si atom is replaced by an Al atom in the 2 × 2 unit cell (the parts of the curves with zero slope) [24]. The $\Delta \gamma_{\left(11\bar{1}\right)}$ dependences are calculated using the value of the AlN/SiC($11\bar{1}$) interface energy for the ideal interface in which one Al atom is replaced by an Si atom in the 2 × 2 unit cell [24].

Calculation the surface energies of polar surfaces and interfaces is a complex problem. As a rule, calculations of these energies are performed using density-functional theory (DFT). The data on the absolute values of the polar AlN (SiC) surface energies and of the AlN/SiC interface energies are rarely reported in the literature. It should also be noted that the obtained values of these energies are approximate and, generally speaking, can be used only for qualitative analysis. Akiyama et al. [24,26] calculated by DFT the surface energies of the polar facets, AlN(0001) (with one N atom located above the “hexagonal channel” in the 2 × 2 unit cell) and AlN($000\bar{1}$) (with the adsorption monolayer of Al), as well as the interface energies of the AlN/SiC(111) and AlN/SiC($\bar{1}\bar{1}\bar{1}$) interfaces (the latter interface is identical to the AlN/SiC($11\bar{1}$) interface) (Figure 7). These values were obtained as functions of the difference $\mu_{Al}-\mu_{Al}^{bulk}$(in eV), where $\mu_{Al}$ is the chemical potential of Al on the AlN surface and $\mu_{Al}^{bulk}$ is the chemical potential of bulk Al. The values of $\mu_{Al}-\mu_{Al}^{bulk}$ belong to the interval from E_AlN_ to 0, where E_AlN_ = −2.8 eV is the AlN formation enthalpy. The surface energies of the ideal SiC(111) and SiC($\bar{1}\bar{1}\bar{1}$) surfaces terminated by Si and C atoms, respectively, were calculated by Abavare et al. [27] using DFT: 2.856 J/m^2^ and 3.065 J/m^2^ for unrelaxed surfaces; 1.830 J/m^2^ and 2.720 J/m^2^ for relaxed surfaces. The surface energies of SiC(111) and SiC($\bar{1}\bar{1}\bar{1}$) were calculated assuming that an excess of Si and C atoms, respectively, is formed on the surfaces. The calculated values of Δγ, which determine the nucleation mechanism of AlN islands on SiC, are shown in Figure 7 as a function of the chemical potential difference $\mu_{Al}-\mu_{Al}^{bulk}.\;$ The graphs in Figure 7 suggest how to determine the growth conditions under which the nucleation rates of the AlN islands on SiC(111) and SiC($11\bar{1}$) are either the same or different. For example, the condition $\Delta \gamma_{\left(111\right)}\;$=$\;\;\Delta \gamma_{\left(11\bar{1}\right)}$ is fulfilled if $\mu_{Al}-\mu_{Al}^{bulk}\;\approx$ −1.4 eV (or $\mu_{Al}-\mu_{Al}^{bulk}\;\approx$ −1 eV when using the surface energies of unrelaxed SiC surfaces). Thus, the nucleation rates of three-dimensional islands on the SiC(111) and SiC($11\bar{1}$) facets are equal if the intermediate values of the chemical potential of Al on the AlN surface are maintained, i.e., $\mu_{Al}-\mu_{Al}^{bulk}\;\approx \;\mu_{N}-\mu_{N}^{bulk}$. The AlN growth rate should be more intensive in the SiC[$11\bar{1}$] direction ($\Delta \gamma_{\left(11\bar{1}\right)}\;$< $\gamma_{\left(111\right)}$) in the Al-rich condition and vice versa, in the N-rich condition, the AlN.

Growth primarily occurs in the SiC[111] direction ($\Delta \gamma_{\left(111\right)}$ < $\;\Delta \gamma_{\left(11\bar{1}\right)}$). Since the V/III ratio on the substrate is determined by that in the gas phase, the Al-rich condition can be interpreted as a result of a small V/III ration in the gas phase. It also follows from Figure 7 that, in the Al-rich condition, the value of $\Delta \gamma_{\left(11\bar{1}\right)}$ may become negative. Then, a two-dimensional growth mode of the AlN layer may become energetically favorable, as follows from Equations (4) and (6).

It is important to note that the thermodynamic analysis is performed without considering surface reconstructions involving foreign adsorbed particles in the system (e.g., H atoms). If the AlN growth proceeds in the atmosphere of H_2_ (the carrier gas instead of Ar), an excess of H may be formed on the surface. The presence of H will change the value of the surface energies of the SiC(111) and SiC($11\bar{1}$) facets and, as shown in [28], their values will be close to each other. According to the calculation [26], in the presence of adsorbed hydrogen, the surface energy of polar AlN surfaces will also change. In the H-rich condition, the surface energy of AlN(0001) becomes less than the surface energy of AlN($000\bar{1}$) over the entire range of Al chemical potential. Consequently, in this case, the growth rate of AlN on the SiC($11\bar{1}$) facets will be higher than that on the SiC(111) facets. From the performed analysis, it follows that, in the experimentally observed growth of AlN on SiC/Si(110), the condition $\mu_{Al}-\mu_{Al}^{bulk}\;\approx \mu_{N}-\mu_{N}^{bulk}$ is approximately satisfied since the numbers of hexagonal AlN crystals turned in two different directions are comparable quantities (differ by factor 2). Note that the ration of this numbers is also determined by the available surface area of the SiC(111) and SiC($11\bar{1}$) facets formed before the AlN film growth. Without considering the surface reconstructions, one can show using the Wulff construction that due to the higher energy of the SiC($11\bar{1}$) facets [27,28], their area should be smaller; however, in the H-rich condition, the areas of the SiC(111) and SiC($11\bar{1}$) facets will have close values. Thus, replacement of the Ar carrier gas with the H_2_ carrier gas can lead to a change in the growth mechanism of semipolar AlN layers on the SiC/Si(110) substrates. Moreover, in the H-rich condition, the chemical reaction of AlN formation should differ from the reaction (2) [5]. It is also important to note that, in the presence of foreign atoms in the system, it may become energetically favorable to nucleate AlN crystals of one polarity. The presence of two polarities of AlN crystals will be verified in further studies.

The proposed model shows that, in principle, it is possible to control the relative number of hexagonal crystals growing in two different directions by changing the V/III ratio in the gas phase and, consequently, by changing this ration on the substrate.

## 5. Conclusions

In conclusion, the growth mechanism of AlN films by HVPE on the hybrid SiC/Si(110) substrates has been investigated. The structural and morphological properties of AlN films and SiC buffer layers were studied by using XRD, SEM, RHEED and Raman spectroscopy. The crystallographic relations between AlN film and the substrate were determined, and it was found that the AlN film growth occurs by the merging of the hexagonal AlN microcrystals growing in two preferential directions. It was shown that the hybrid SiC/Si(110) substrates can be used as templates for the synthesis of semipolar AlN($10\bar{1}3$) layers by HVPE. The growth of semipolar AlN on the SiC/Si(110) was investigated both experimentally and theoretically. The theoretical results concerning the nucleation mechanism of AlN are fully consistent with the experimental data. Based on the investigation of the structure and morphology of AlN layers, a crystallographic model explaining the presence of two orientations of the AlN film on SiC/Si(110) was proposed. It was found that it is possible to change the ratio of the number of crystals growing in two directions by changing the V/III ratio in the gas phase. The proposed model also explains the experimentally observed relationship between the crystallographic orientations of AlN and SiC. The presented study is important both from a purely scientific point of view, since the nucleation mechanisms of the AlN($10\bar{1}3$) layers on the hybrid SiC/Si(110) substrates have been considered for the first time, and also for future practical applications. Semipolar AlN layers can be used not only as a material for microelectronics, but they can also act as substrates for the growth of compounds such as AlGaN and GaN.

## Data Availability

Data is contained within the article.

## References

[1] Yamashita H., Fukui K., Misawa S., Yoshida S. (1979). Optical Properties of AlN Epitaxial Thin Films in the Vacuum Ultraviolet Region. J. Appl. Phys..

[2] Kukushkin S.A., Osipov A.V., Bessolov V.N., Medvedev B.K., Nevolin V.K., Tcarik K.A. (2008). Substrates for Epitaxy of Gallium Nitride: New Materials and Techniques. Rev. Adv. Mater. Sci..

[3] Masui H., Nakamura S., DenBaars S.P., Mishra U.K. (2010). Nonpolar and Semipolar III-Nitride Light-Emitting Diodes: Achievements and Challenges. IEEE Trans. Electron. Devices.

[4] Takeuchi T., Amano H., Akasaki I. (2000). Theoretical Study of Orientation Dependence of Piezoelectric Effects in Wurtzite Strained GaInN/GaN Heterostructures and Quantum Wells. Jpn. J. Appl. Phys..

[5] Bessolov V.N., Konenkova E.V., Kukushkin S.A., Osipov A.V., Rodin S.N. (2014). Semipolar Gallium Nitride on Silicon: Technology and Properties. Rev. Adv. Mater. Sci..

[6] Abe Y., Komiyama J., Isshiki T., Suzuki S., Yoshida A., Ohishi H., Nakanishi H. (2008). Semipolar Nitrides Grown on Si(001) Offcut Substrates with 3C-SiC Buffer Layers. Mater. Sci. Forum.

[7] Jo M., Hirayama H. (2018). Effects of Ga Supply on the Growth of (11-22) AlN on *m*-Plane (10-10) Sapphire Substrates. Phys. Status Solidi.

[8] Shen X.-Q., Kojima K., Okumura H. (2020). Single-Phase High-Quality Semipolar (10-13) AlN Epilayers on m-Plane (10–10) Sapphire Substrates. Appl. Phys. Express.

[9] Bessolov V., Kalmykov A., Konenkov S., Konenkova E., Kukushkin S., Myasoedov A., Osipov A., Panteleev V. (2017). Semipolar AlN on Si(100): Technology and Properties. Microelectron. Eng..

[10] Li X., Zhao J., Liu T., Lu Y., Zhang J. (2021). Growth of Semi-Polar (101-3) AlN Film on M-Plane Sapphire with High-Temperature Nitridation by HVPE. Materials.

[11] Bessolov V., Kalmykov A., Konenkova E., Kukushkin S., Myasoedov A., Poletaev N., Rodin S. (2017). Semipolar AlN and GaN on Si(100): HVPE Technology and Layer Properties. J. Cryst. Growth.

[12] Kukushkin S.A., Osipov A.V., Redkov A.V., Sharofidinov S.S. (2020). Epitaxial Growth of Bulk Semipolar AlN Films on Si(001) and Hybrid SiC/Si(001) Substrates. Tech. Phys. Lett..

[13] Lahourcade L., Bellet-Amalric E., Monroy E., Abouzaid M., Ruterana P. (2007). Plasma-Assisted Molecular-Beam Epitaxy of AlN(112-2) on m Sapphire. Appl. Phys. Lett..

[14] Ueno K., Kobayashi A., Ohta J., Fujioka H., Amanai H., Nagao S., Horie H. (2009). Room Temperature Growth of Semipolar AlN (1-102) Films on ZnO (1-102) Substrates by Pulsed Laser Deposition. Phys. Status Solidi Rapid Res. Lett..

[15] Kukushkin S.A., Osipov A.V. (2014). Theory and Practice of SiC Growth on Si and Its Applications to Wide-Gap Semiconductor Films. J. Phys. D Appl. Phys..

[16] Kukushkin S.A., Osipov A.V. (2021). Nanoscale Single-Crystal Silicon Carbide on Silicon and Unique Properties of This Material. Inorg. Mater..

[17] Kalinkin I.P., Kukushkin S.A., Osipov A.V. (2018). Effect of Chemical Treatment of a Silicon Surface on the Quality and Structure of Silicon-Carbide Epitaxial Films Synthesized by Atom Substitution. Semiconductors.

[18] Kukushkin S.A., Osipov A.V. (2022). Dielectric Function and Magnetic Moment of Silicon Carbide Containing Silicon Vacancies. Materials.

[19] Goldberg Y., Levinshtein M.E., Rumyantsev S.L., Shur M.S. (2001). Properties of Advanced Semiconductor Materials: GaN, AIN, InN, BN, SiC, SiGe.

[20] Kuball M. (2001). Raman Spectroscopy of GaN, AlGaN and AlN for Process and Growth Monitoring/Control. Surf. Interface Anal..

[21] Freitas J.A., Culbertson J.C., Mastro M.A., Kumagai Y., Koukitu A. (2012). Structural and Optical Properties of Thick Freestanding AlN Films Prepared by Hydride Vapor Phase Epitaxy. J. Cryst. Growth.

[22] Jindal V., Shahedipour-Sandvik F. (2009). Theoretical Prediction of GaN Nanostructure Equilibrium and Nonequilibrium Shapes. J. Appl. Phys..

[23] Markov I.V. (2003). Crystal Growth for Beginners.

[24] Akiyama T., Nakane H., Nakamura K., Ito T. (2016). Effective Approach for Accurately Calculating Individual Energy of Polar Heterojunction Interfaces. Phys. Rev. B.

[25] Lu J., Chen J.-T., Dahlqvist M., Kabouche R., Medjdoub F., Rosen J., Kordina O., Hultman L. (2019). Transmorphic Epitaxial Growth of AlN Nucleation Layers on SiC Substrates for High-Breakdown Thin GaN Transistors. Appl. Phys. Lett..

[26] Akiyama T., Nakane H., Uchino M., Nakamura K., Ito T. (2018). Structures and Polarity of III-Nitrides: Phase Diagram Calculations Using Absolute Surface and Interface Energies. Phys. Status Solidi.

[27] Abavare E.K.K., Iwata J.-I., Yaya A., Oshiyama A. (2014). Surface Energy of Si(110)- and 3C-SiC(111)-Terminated Surfaces. Phys. Status Solidi.

[28] Sambonsuge S., Nikitina L.N., Hervieu Y.Y., Suemitsu M., Filimonov S.N. (2014). Silicon Carbide on Silicon (110): Surface Structure and Mechanisms of Epitaxial Growth. Russ. Phys. J..

